# Reactivation of inactive X chromosome in buccal smear of carcinoma of breast

**DOI:** 10.4103/0971-6866.42320

**Published:** 2008

**Authors:** Prashant E. Natekar, Fatima M. DeSouza

**Affiliations:** Department of Anatomy, Goa Medical College, Bambolim, Goa, India

**Keywords:** Barr body, breast cancer, buccal smear, sex chromatin

## Abstract

Buccal mucosal smears of 100 female patients of carcinoma of breast were compared with 100 controls matched accordingly. The frequency of Barr bodies was significantly lower in carcinoma of breast patients (menstruating and menopausal women) *P* < 0.001 when compared with controls indicating reactivation of the inactive X chromosome.

## Introduction

In somatic cells of normal females, one X chromosome is inactivated. The Barr body, a chromatin mass visible in the interphase, represents the late replicating inactive X. The number of Barr bodies is always one less than the total number of X chromosomes per cell. Inactivation is believed to occur early in embryonic life and to be completed by the end of the first week of development. There is random inactivation of the maternally and paternally derived X chromosomes in any one female somatic cell.[1] Twenty years after the paper of Barr and Bertram, the fluorescent (F) body, or Y chromatin was discovered in interphase nuclei.[2]

The frequency of sex chromatin is deceased during pregnancy as well as in women on oral contraceptives.[3] Low frequency of sex chromatin was observed in newborn females and their mothers on the first postpartum day, increased gradually on second and third day, which stabilized on fifth day and became similar in both mothers and the children.[4]

The process of inactivation is incompletely understood. This process is under the control of inactivation center, located at Xq13. XIST, a gene which is transcribed from the inactive X is both necessary and sufficient for initiation and propagation of X inactivation does so by coating the inactive X. It is not clear whether XIST plays a role in maintenance of inactivation, which likely occurs through differential methylation.[5] Reactivation of X chromosome was also observed whenever the body was under physiological stress.[6] Low frequency suggestive of reactivation of inactive X chromosome is associated with malignancy[7–9] and is confirmed by enhanced G-6PD activity, and low X chromatin frequency in cancer tissue leads to reactivation of inactive X chromatin.[10]

The present study is to find the frequency of X chromatin in carcinoma of breast patients so as to confirm whether there is reactivation of inactive X chromosome by comparing with that of the control.

## Materials and Methods

This study was carried out in 100 female patients of carcinoma of breast attending the Radiotherapy Department of Goa Medical College, Bambolim, Goa. Both the cases of carcinoma of breast and normal controls were selected randomly for inclusion in this study in the age group between 35 and 60. The diagnosis of these patients was confirmed by histopathologic biopsy. These patients were divided into two groups. Group I consisted of carcinoma of breast who had no history of any other genetic disorder or heredity diseases. They were matched with 100 controls (Group II) having no family history of breast cancer or any other inheritable diseases. Buccal mucosal smear of all the subjects after a good mouth with water was obtained with a metal spatula from the inner surface of the cheek after applying firm pressure. The scrapings of buccal mucosa were spread on a clean slide. The slides were numbered and immersed in 95% ethyl alcohol (fixative) to prevent from drying. The smears were stained with carbol fuschin.[11] In each slide, 100 cells having vesicular nuclei with distinguished border were counted for this study. It was ensured that the sex chromatin was located at the inner side of the nuclear membrane. All the other cells lacking X chromatin were considered as negative.

The results were evaluated by using the Student *t*-test to determine the frequency of X chromatin.

## Results

In our present study, it was observed that the frequency of X chromatin in carcinoma of breast patients (menstruating as well as menopausal) was significantly decreased *P* < 0.0001 as compared to that of the controls as shown in Table 1.

**Table 1:** X chromatin frequency in carcinoma of breast with that of the control

Groups	Mean	SD	95% CI (LL)	95% CI (UL)	*t* Value	*P* value
Ca breast (Group I)	6.08	2.35	1.47	13.13	37.26	0.0001
Control (Group II)	19.6	2.76	14.18	27.89		

df = 198; CI - confidence interval; SD - standard deviation; LL - lower limit, UL - upper limit

## Discussion

Although higher frequency of X chromatin has been reported in breast cancer patients,[12] no significant differences of frequency of X chromatin were obtained between menstruating breast cancer patients and menstruating normal women.[13–15]

However, in our present study the frequency of X chromatin in breast cancer is significantly low in menstruating as well as menopausal patients as compared to that of the control, which contradicts the various studies mentioned earlier.

This study suggests that low frequency of inactive X chromatin indicates reactivation of inactive X chromosome.
